# Supplementary material for: Anionic nanoplastic exposure induces endothelial leakiness
Source: Nat Commun. 2022 Aug 13;13:4757. doi: 10.1038/s41467-022-32532-5 (PMC9376074; doi:10.1038/s41467-022-32532-5)
Supplement: Supplementary file 1 — Supplementary Information [file 41467_2022_32532_MOESM1_ESM.pdf]

Supplementary Information

Anionic Nanoplastic Exposure Induces Endothelial Leakiness

Wei and Li *et al.*

List of Content

Supplementary Figures 1-19

Supplementary Tables 1-3

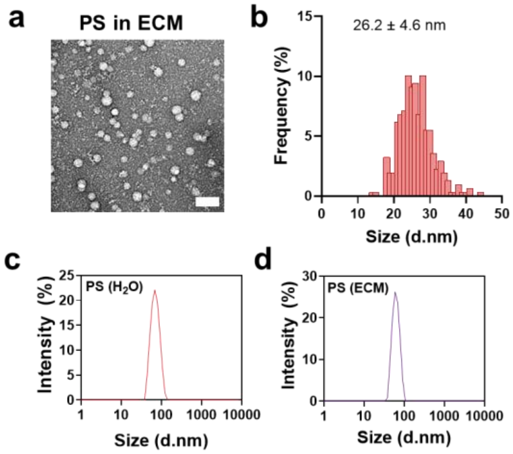

Supplementary Figure 1 Characterisations of polystyrene nanoplastic. a, b TEM imaging of polystyrene (PS) nanoplastic in endothelia cell medium (ECM) and their corresponding size distribution (n=307 nanoparticles examined). Scale bar: 50 nm. **c, d** Hydrodynamic diameter measurements of PS nanoplastic in H_2O and ECM by DLS. Source data are provided as a Source Data file.

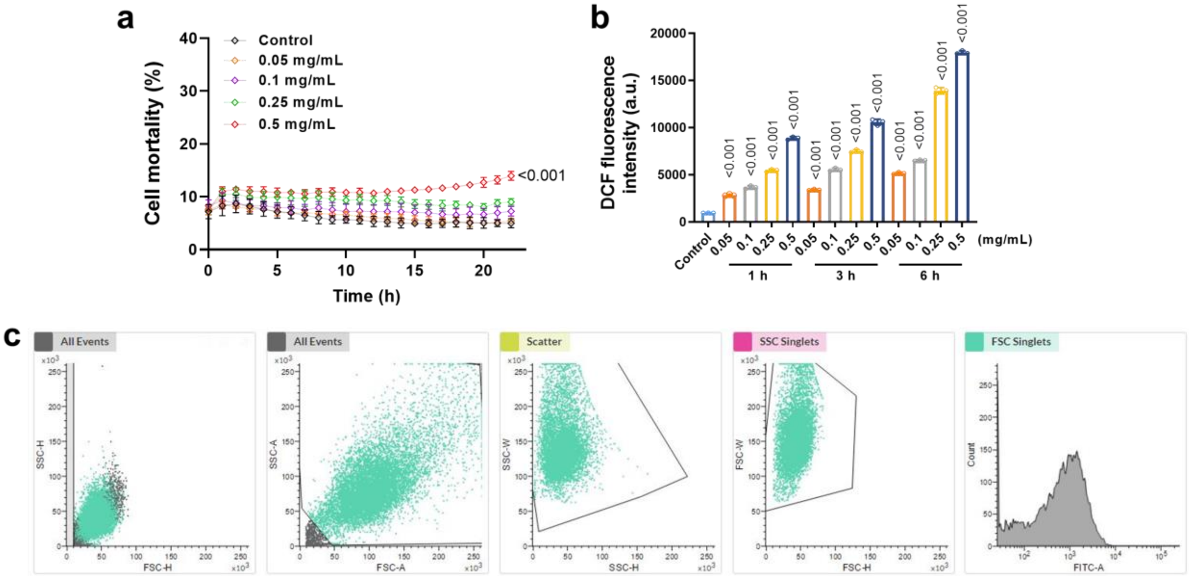

Supplementary Figure 2 Cytotoxicity of PS nanoplastic. a HUVEC cell mortality exposed to PS nanoplastic of different concentrations. Dead cells were stained by propidium iodide (PI). Data are expressed as means ± SD (n=3 biologically independent samples). Statistical analysis was performed through one-way ANOVA followed by Tukey's multiple comparison tests. Compared with control, there were significant differences observed for PS nanoplastic at 0.5 mg/mL at 22 h exposure. *P* value (<0.001 vs control) was inserted in the panel. **b** Generation of reactive oxygen species (ROS) in HUVECs over 1 to 6 h-treatment with PS nanoplastic of different concentrations. All data are represented as the mean ± SD (n=3 biologically independent samples). *P* values were derived from one-way ANOVA followed by Tukey's multiple comparison tests, and inserted in the panel. Compared with control, there were significant differences observed for PS nanoplastic at different concentrations. **c** The FACS sequential gating strategies for ROS generation via the FITC channel. Source data are provided as a Source Data file.

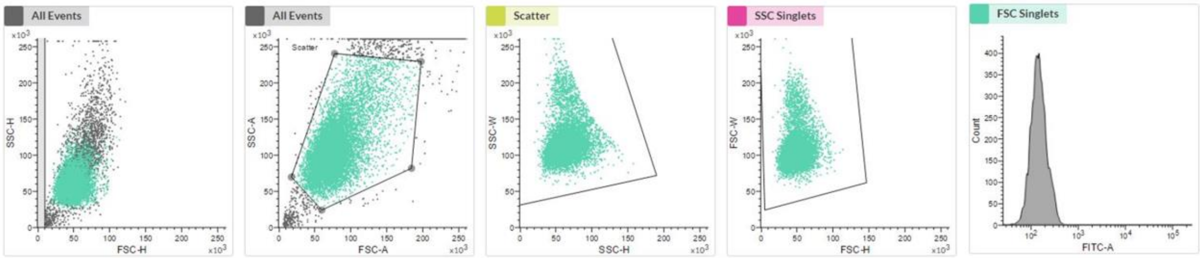

Supplementary Figure 3 The FACS sequential gating strategies for the cellular uptake of PS nanoplastic, which was labelled with green dye and determined via the FITC channel.

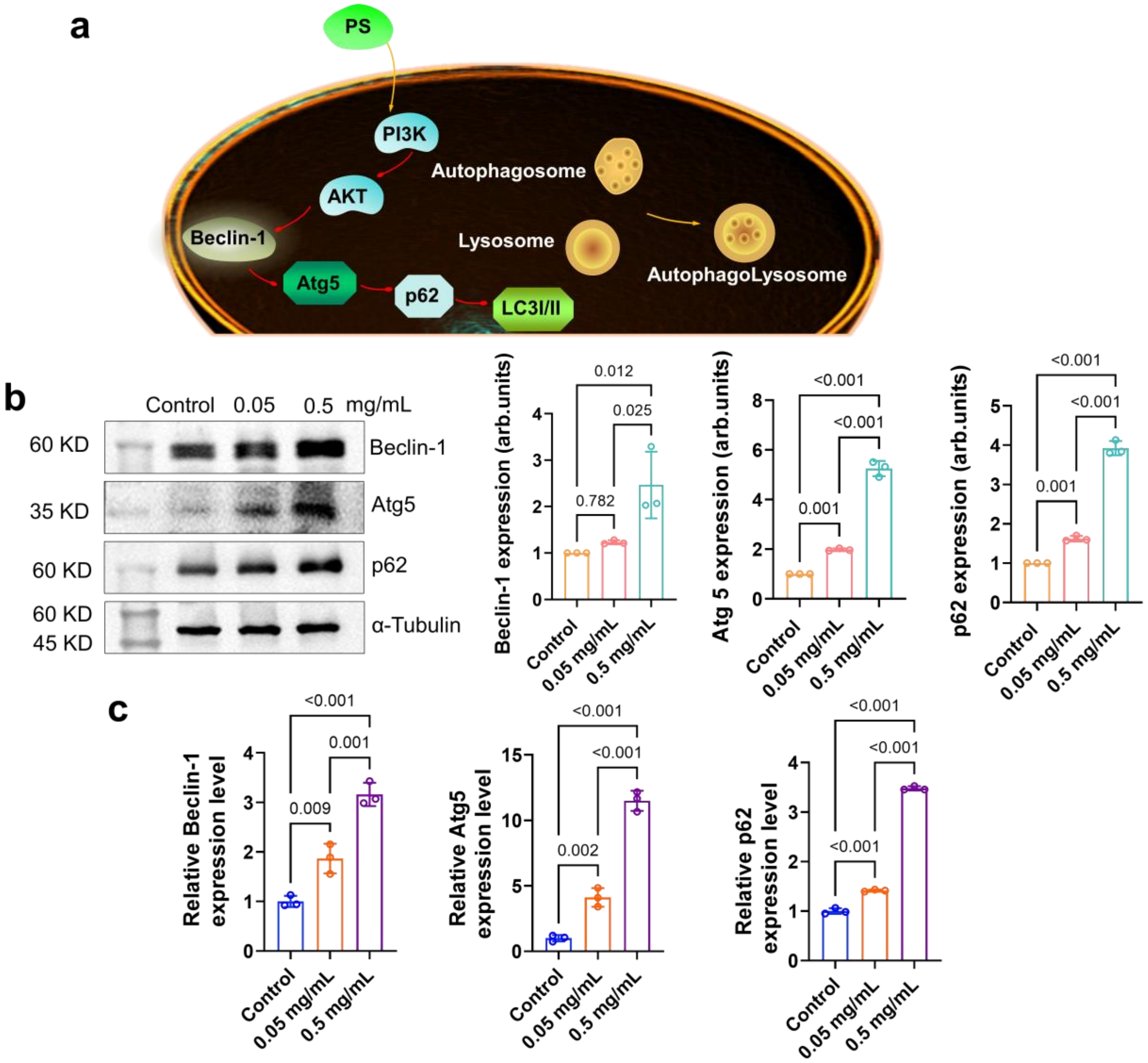

Supplementary Figure 4 Polystyrene nanoplastic-induced autophagy in HUVECs. a PS nanoplastic-induced autophagy signaling pathway in HUVECs. HUVECs were treated with 0.05 or 0.5 mg/mL PS nanoplastic for 6 h. **b** Western blot and semi-quantitative analysis of autophagic proteins. Data are expressed as means ± SD. Biologically independent samples were used (n=3). Statistical analysis was performed through one-way ANOVA followed by Tukey's multiple comparison tests. *P* values were inserted in the panel. **c** RT-qPCR analysis of autophagic genes. Data are expressed as means ± SD. Biologically independent samples were used (n=3). Statistical analysis was performed through one-way ANOVA followed by Tukey's multiple comparison tests. *P* values were inserted in the panel. Source data are provided as a Source Data file.

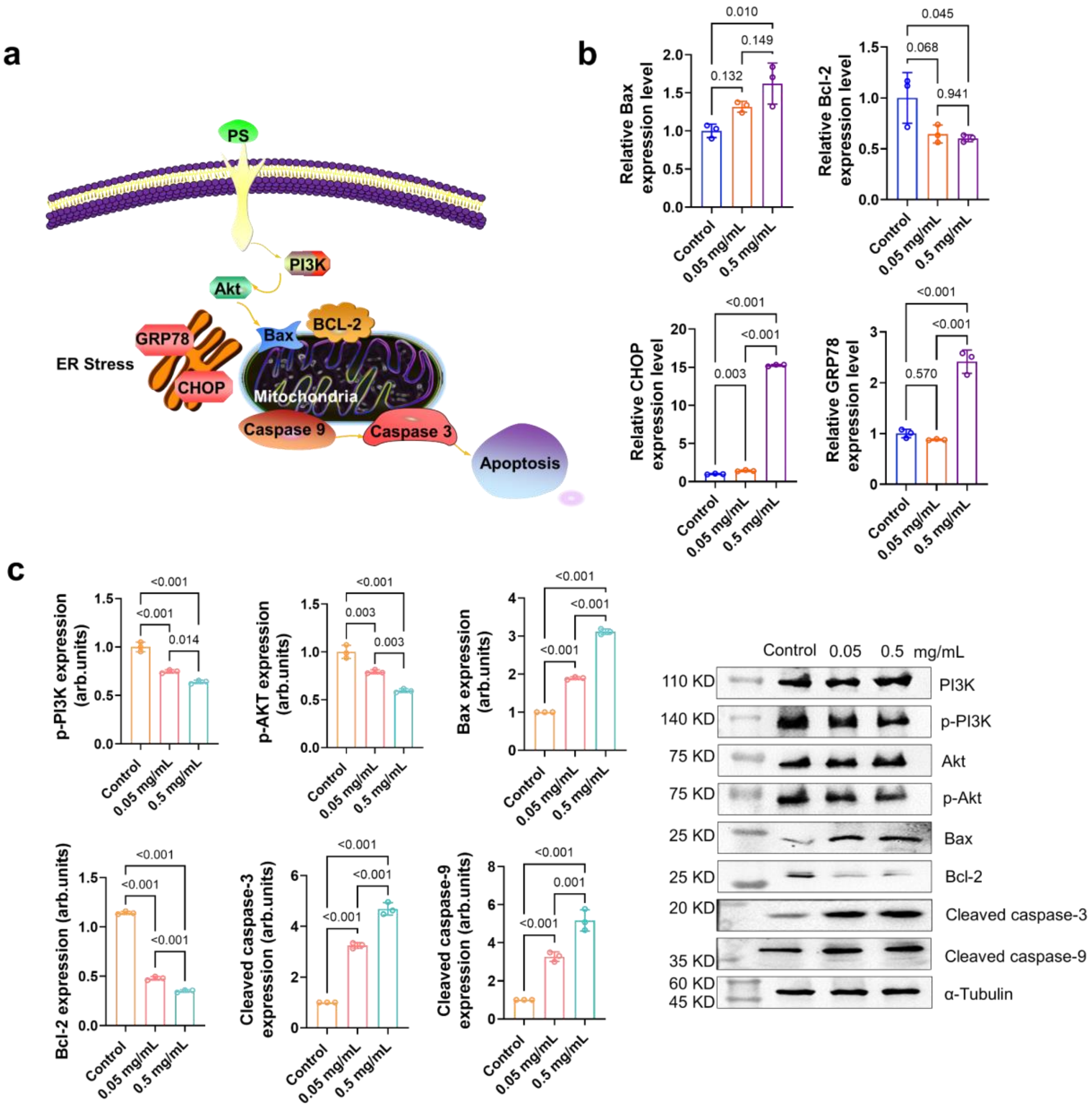

Supplementary Figure 5 Polystyrene nanoplastic-induced apoptosis in HUVECs. a PS nanoplastic-induced apoptosis signaling pathway in HUVECs. HUVECs were treated with 0.05 or 0.5 mg/mL PS nanoplastic for 6 h. **b** RT-qPCR analysis of apoptosis genes. Data are expressed as means ± SD. Biologically independent samples were used (n=3). Statistical analysis was performed through one-way ANOVA followed by Tukey's multiple comparison tests. *P* values were inserted in the panel. **c** Western blot and semi-quantitative analysis of PI3K, AKT and their phosphorylation levels and expression levels of apoptosis proteins. Data are expressed as means ± SD (n=3 biologically independent samples). Statistical analysis was performed through one-way ANOVA followed by Tukey's multiple comparison tests. *P* values were inserted in the panel Source data are provided as a Source Data file.

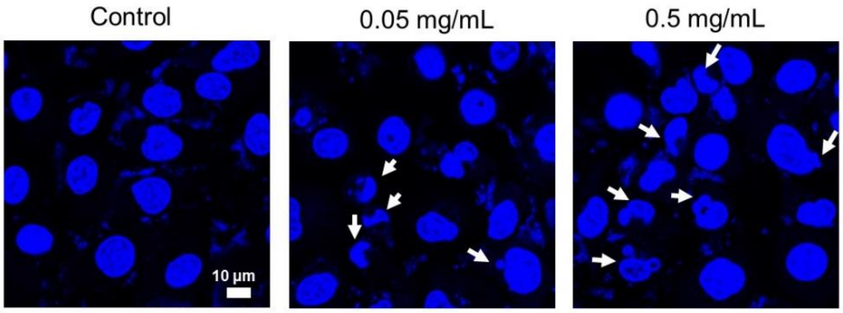

Supplementary Figure 6 DAPI staining of HUVECs exposed to PS nanoplastic. Confocal fluorescence microscopy observed the nucleus morphology of the HUVECs. The white arrows point to apoptotic cells. PS nanoplastic concentration of 0.05 or 0.5 mg/mL and incubation for 6 h (n=3 biologically independent experiments). Scale bar: 10 μm.

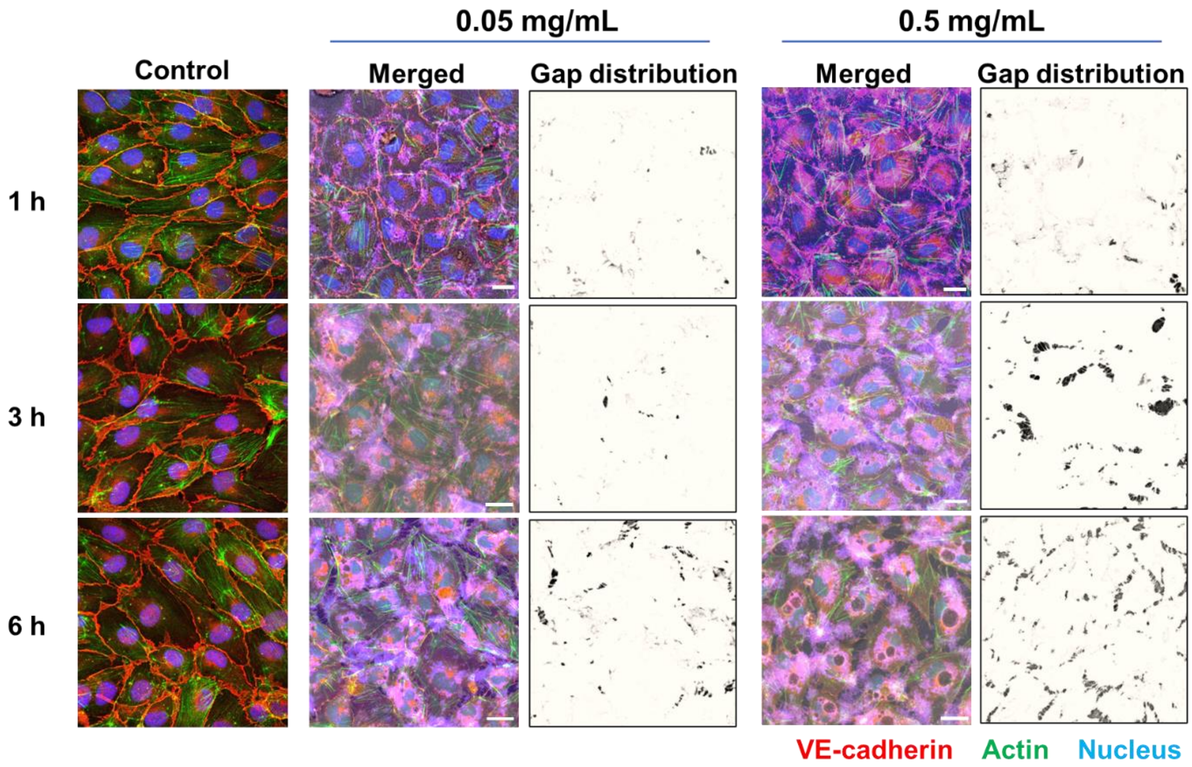

Supplementary Figure 7 Confocal fluorescence microscopy revealed endothelial leakiness with PS nanoplastic of different concentrations (0.05 and 0.5 mg/mL) and for different durations of treatment (1, 3 and 6 h). Rabbit polyclonal anti-VE-cadherin antibody and donkey anti-rabbit Alexa 594 secondary antibody were used to reveal the distribution of VE-cadherins (red). Phalloidin-iFluor 488 and DAPI were used to lable actin (green) and nucleus (blue) (n=3 biologically independent experiments). Scale bars: 20 μm. Gap distribution was analysed using the Trainable Weka Segmentation plugin through ImageJ 1.53c.

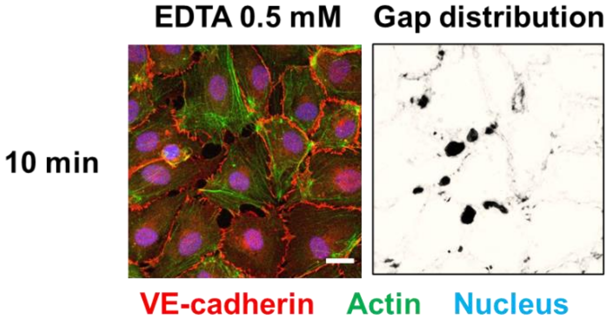

Supplementary Figure 8 Confocal fluorescence microscopy revealed endothelial leakiness in the presence of EDTA 0.5 mM for 10 min. Channels: VE-cadherin (red), actin (green), nucleus (blue) (n=3 biologically independent experiments). Scale bar: 20 μm.

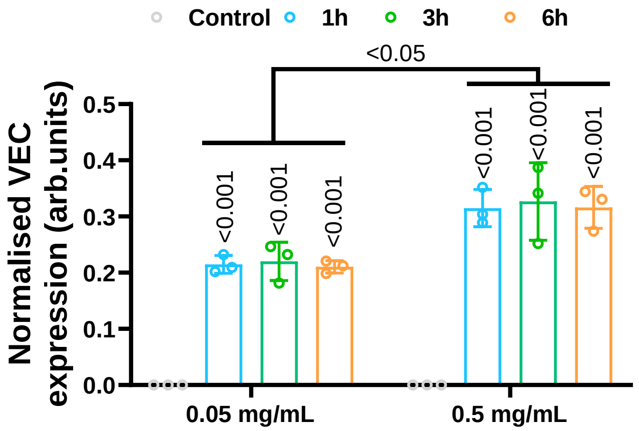

Supplementary Figure 9 Semi-quantitative analysis of Fig. 1f revealed that PS directly bound to adherens junctional homophilic VE–cadherins in a dose-dependent manner. Data are expressed as means ± SD. Biologically independent samples were used (n=3). Statistical analysis was performed through one-way ANOVA followed by Tukey's multiple comparison tests. The derived *P* values between control and sample groups were inserted. Source data are provided as a Source Data file.

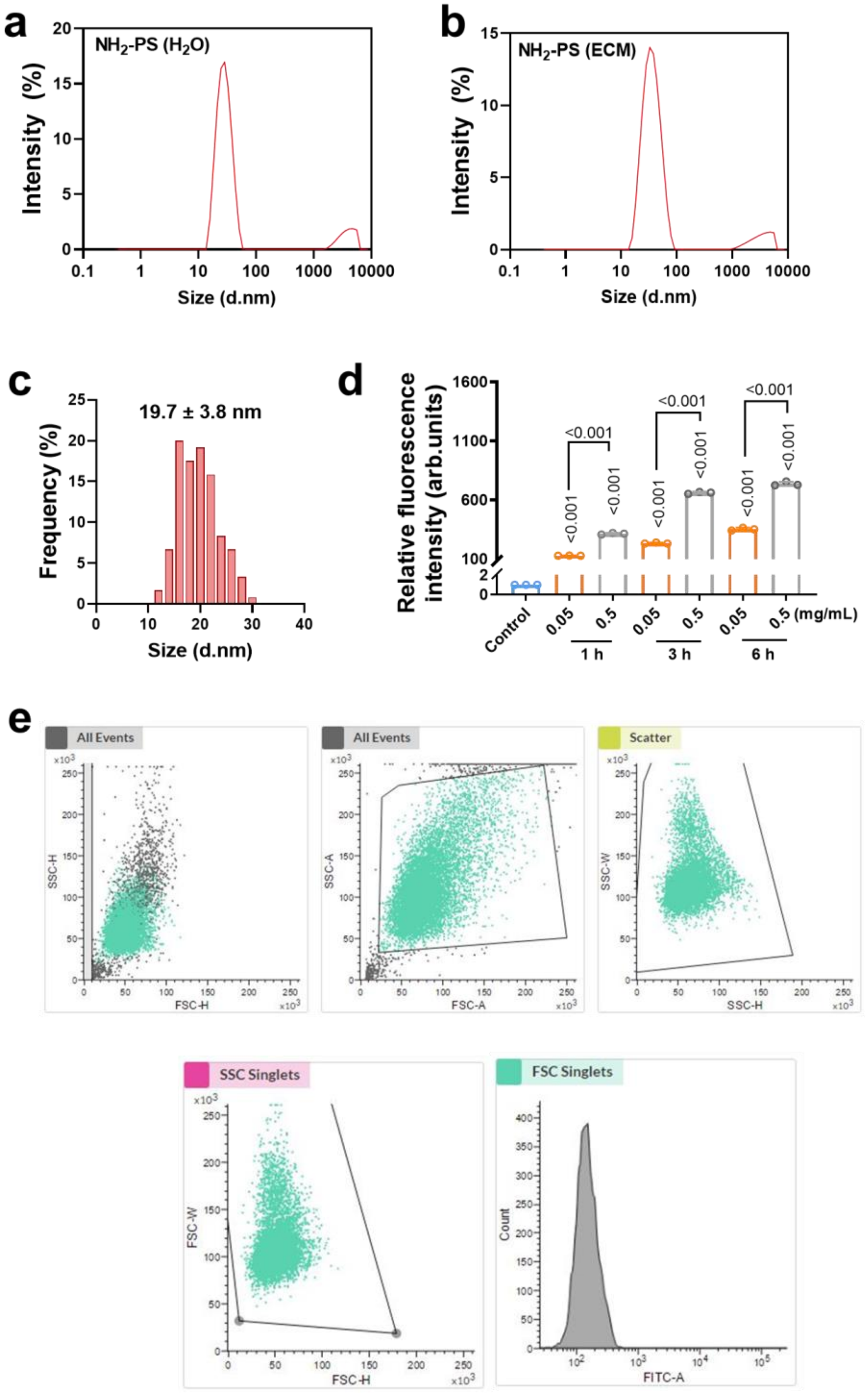

Supplementary Figure 10 Characterisations of NH_2-PS nanoplastic. a, b DLS measurements of NH_2-PS nanoplastic in water and cell media ECM. **c** Corresponding size distribution of the TEM images in **Fig. 3a**. The mean size of NH_2-PS nanoplastic is 19.7 ± 3.8 nm. **d** Cellular uptake of NH_2-PS nanoplastic (0.05 and 0.5 mg/mL) in HUVECs at different time points (1, 3 and 6 h). The relative fluorescence intensities were normalized by the intensities of the same concentrations of PS nanoplastic. Data are expressed as means ± SD. Biologically independent samples were used (n=3). Statistical analysis was performed through two-way ANOVA followed by Tukey's multiple

comparison tests. The derived *P* values between control and sample groups were inserted. **e** The FACS sequential gating strategies for the cellular uptake of NH_2-PS nanoplastic, which was labelled with green dye and determined via the FITC channel. Source data are provided as a Source Data file.

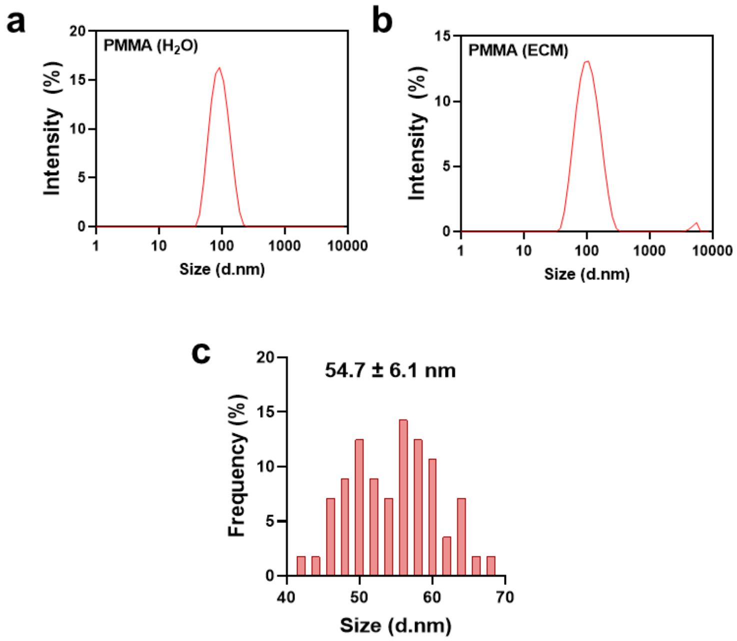

Supplementary Figure 11 Characterisations of PMMA nanoplastic. a, b DLS measurements of PMMA nanoplastic in water and cell medium ECM. **c** Corresponding size distribution of TEM images in **Fig. 3a**. The mean size of PMMA nanoplastic is 54.7±6.1 nm. Source data are provided as a Source Data file.

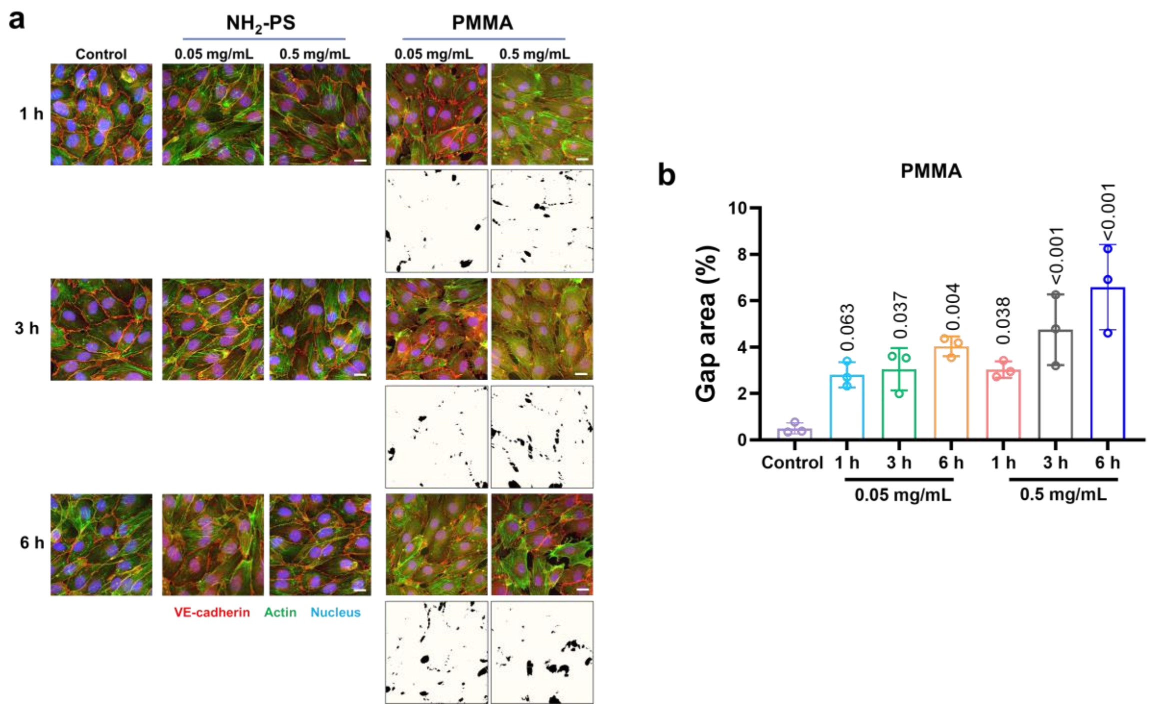

Supplementary Figure 12 PMMA nanoplastic induced endothelial leakiness in HUVECs. a Confocal fluorescence microscopy revealed endothelial leakiness induced by PMMA nanoplastic and NH_2-PS nanoplastic of different concentrations (0.05 and 0.5 mg/mL) and upon 1, 3 and 6 h treatments. Scale bar: 20 µm. Gap distribution was analysed using the Trainable Weka Segmentation plugin through ImageJ. **b** Semi-quantitative analysis of gaps area was performed by ImageJ software according to panel a. Data are expressed as means ± SD (n=3 biologically independent samples). Statistical analysis was performed through one-way ANOVA followed by Tukey's multiple comparison tests. The derived *P* values between control and sample groups were inserted. Source data are provided as a Source Data file.

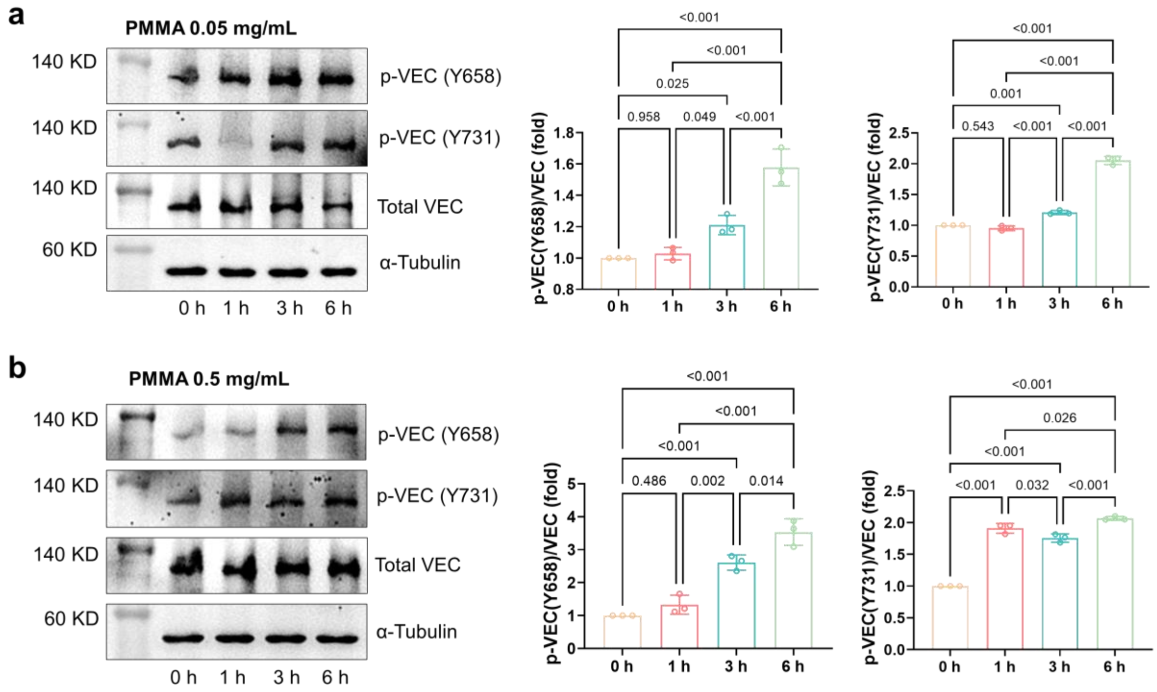

Supplementary Figure 13 Western blot and semi-quantitative analysis of VE-cadherin expression with PMMA nanoplastic. HUVECs were exposed to PMMA nanoplastic at 0.05 mg/mL (**a**) and 0.5 mg/mL (**b**) for different times (1, 3 and 6 h). Protein levels were standardised by comparison with α-Tubulin. Data are expressed as means ± SD. Biologically independent samples were used (n=3). Statistical analysis was performed through one-way ANOVA followed by Tukey's multiple comparison tests. The derived *P* values were inserted. Source data are provided as a Source Data file.

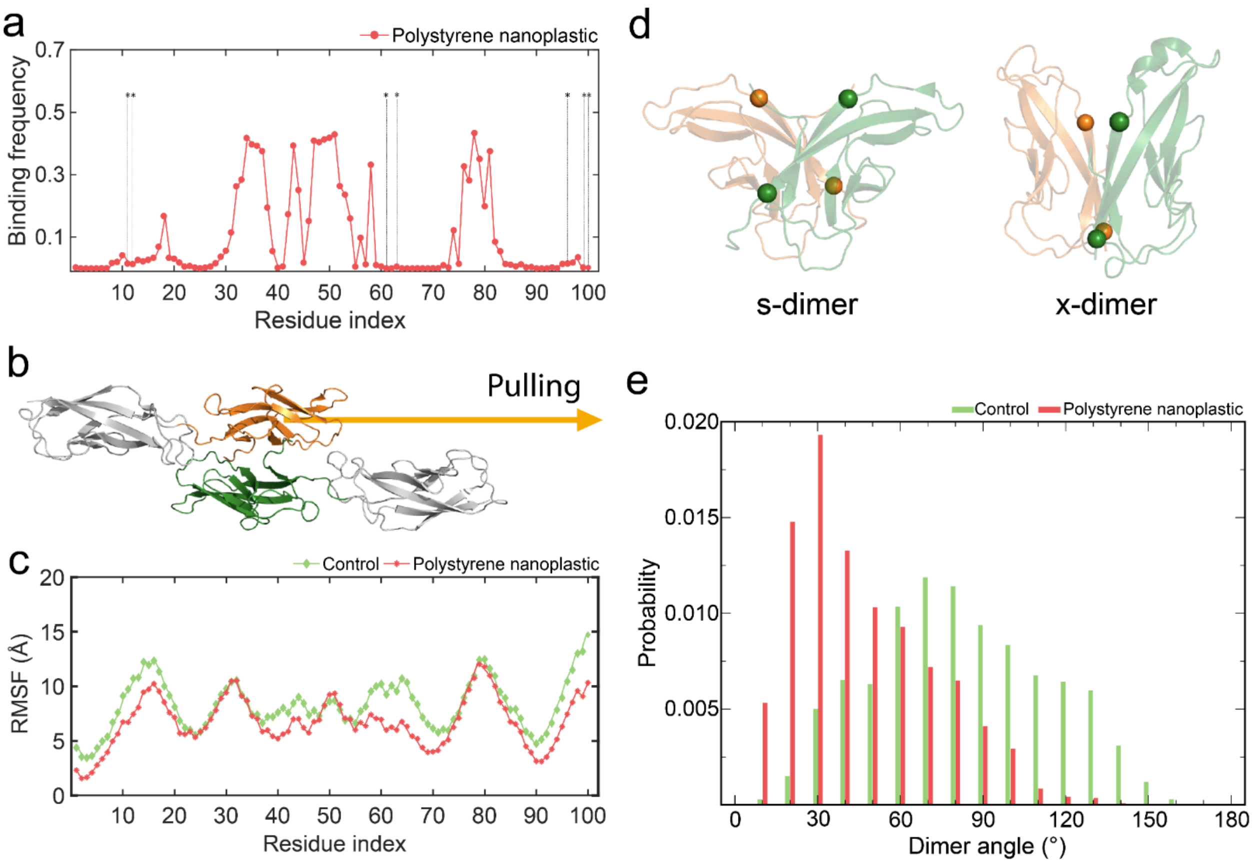

Supplementary Figure 14 Binding frequency and dimer analysis after discrete molecular dynamics (DMD) simulations. a Binding frequency of a PS nanoplastic with a cadherin dimer. The residues coordinating the Ca^{2+} ions are marked with stars (*). **b** Schematic of steered discrete molecular dynamics (sDMD) simulation. Gray color represents the EC2 domains. Green and orange indicate the immobilised and flexible domains of the EC1 dimer, respectively. **c** Root mean square fluctuation (RMSF) of flexible domain from the EC1 cadherin dimer with and without the PS nanoplastic. **d** Structures of different states of the EC1 cadherin dimer. Intermediate and stable states of the dimer known as x-dimer and s-dimer, respectively. **e** Inter dimer angle distribution of the cadherin dimer with and without the PS nanoplastic. For angle distribution and RMSF, first 30 ns of sDMD simulation under 0 pN forces were considered for the analysis.

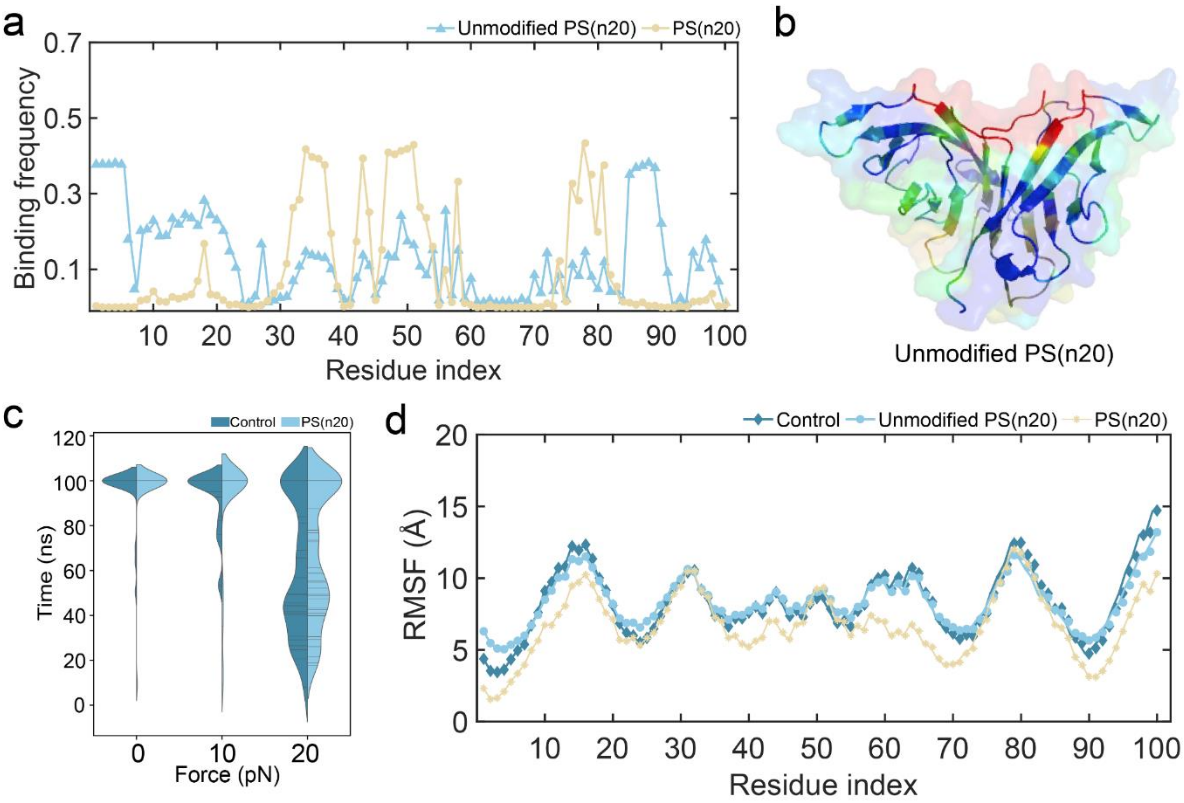

Supplementary Figure 15 Binding and sDMD simulation results comparison between unmodified PS nanoplastic and carboxylated PS nanoplastic. a Binding frequency of unmodified PS and carboxylated PS nanoplastics with the cadherin dimer. **b** Coloured binding frequency on the surface of the dimer complexed with unmodified PS nanoplastics. **c** Violin plot result of the cadherin with and without unmodified PS nanoplastic. **d** Root mean square fluctuation (RMSF) analysis of the cadherin dimer without and with PS nanoplastics.

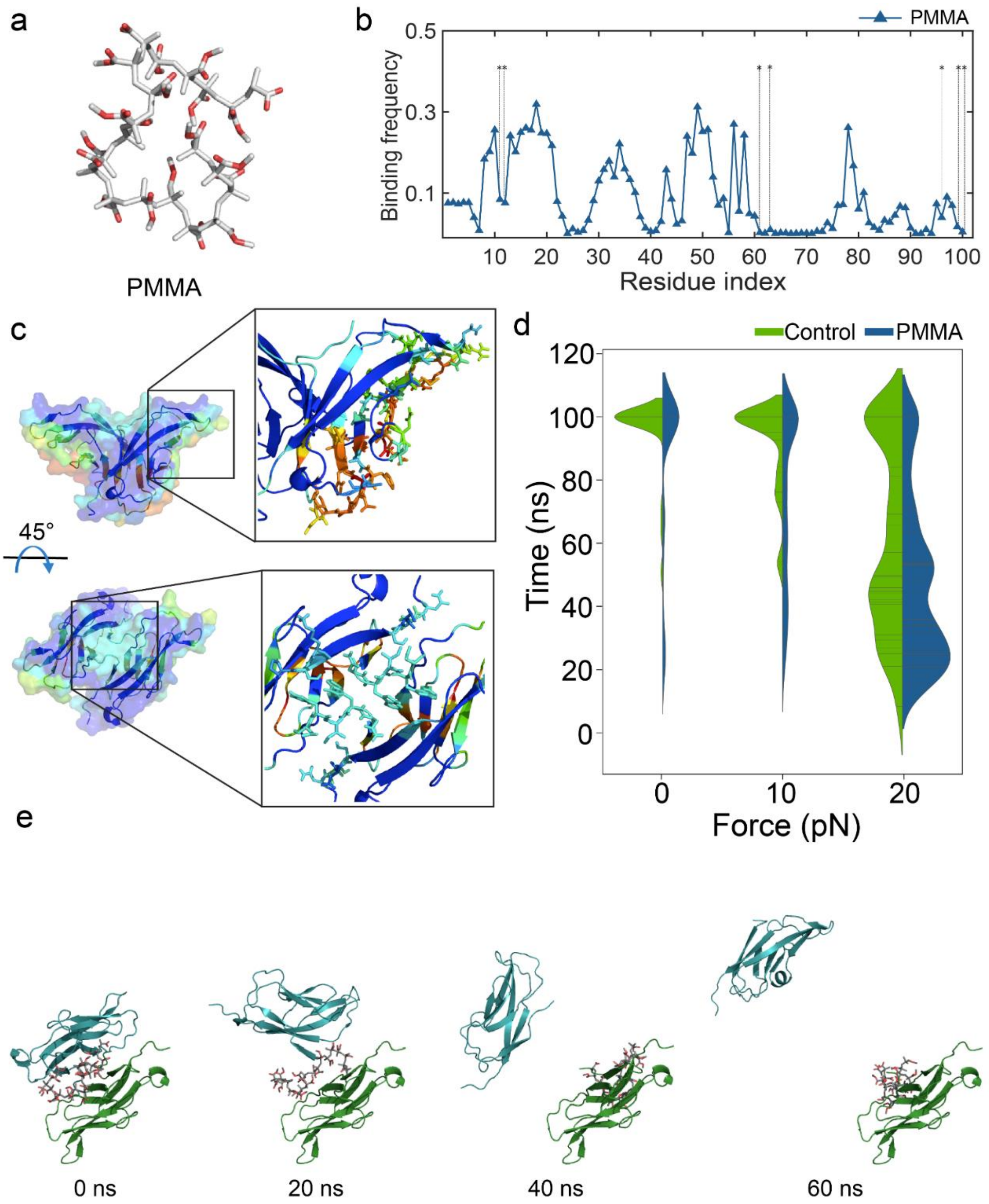

Supplementary Figure 16 DMD simulations of a VE-cadherin dimer with poly(methyl methacrylate) (PMMA). **a** Structure of PMMA 20mer after 50 ns equilibrium DMD simulation. The methyl groups of a subset of the repeating units were removed to render the nanoplastic negatively charged. **b** Binding frequency of the PMMA nanoplastic with the cadherin dimer after 50 ns binding DMD simulation. **c** Coloured binding frequency on the surface of the dimer complexed with PMMA nanoplastic. Blue and red colours indicate low to high binding frequency, respectively. Upper and lower figures were represented from front and bottom views, respectively. Enlarged upper and low panels illustrate details of PMMA nanoplastic binding with the dimer. **d** Violin plots of the dimer complexed with PMMA nanoplastic. **e** An alternative dimer dissociation pathway due to competitive binding of PMMA with one of the monomers under 0 pN of pulling.

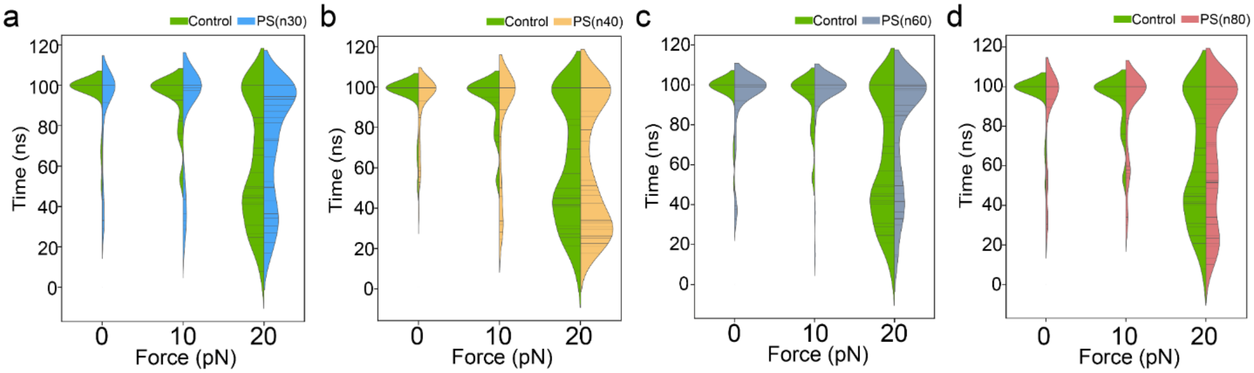

Supplementary Figure 17 Violin plots of cadherin binding with different sizes of PS nanoplastics after 100 ns sDMD simulations. Different sizes of PS nanoplastics such as **a** 30-mer, **b** 40-mer, **c** 60-mer, and **d** 80-mer were employed.

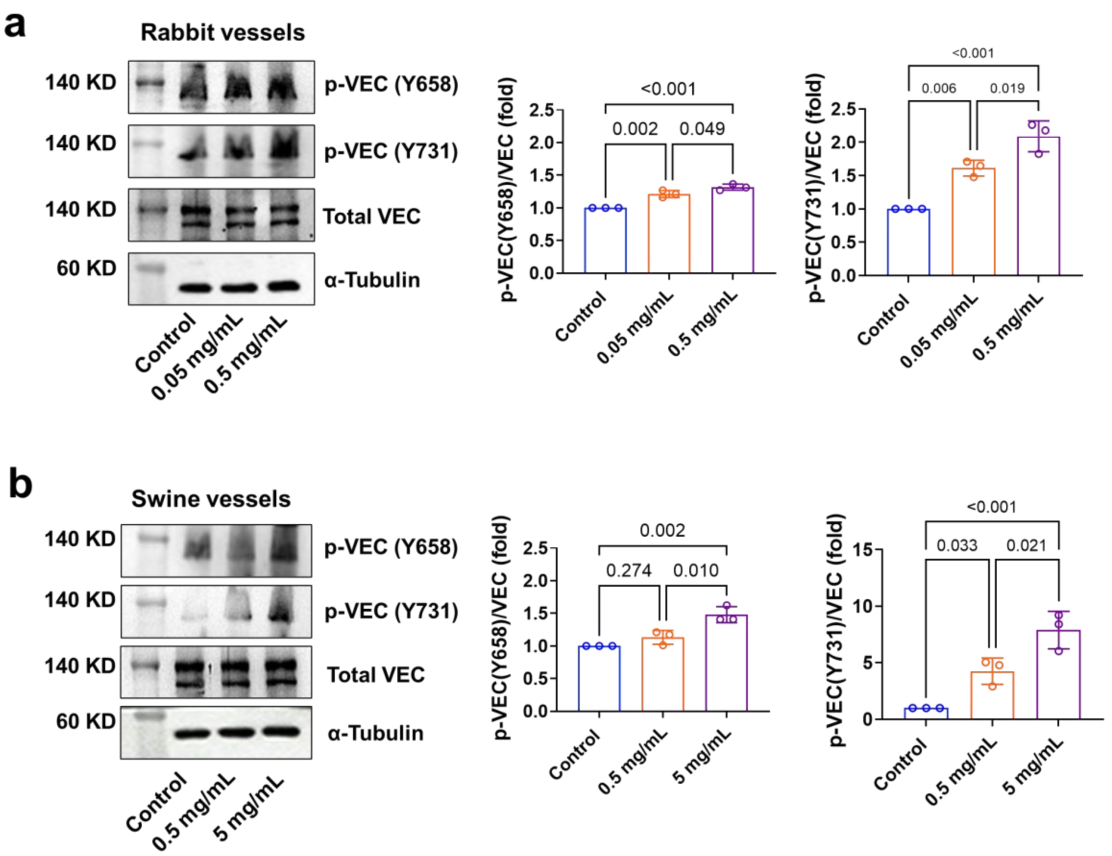

Supplementary Figure 18 Western blot and semi-quantitative analysis of VE-cadherin expression in rabbit (a) and swine (b) vessels after the transwell assay *ex vivo* with polystyrene nanoplastic. Protein levels were standardised by comparison with α-Tubulin. Data are expressed as means ± SD. Biologically independent samples were used (n=3). Statistical analysis was performed through one-way ANOVA followed by Tukey's multiple comparison tests. The derived *P* values between control and sample groups were inserted. Source data are provided as a Source Data file.

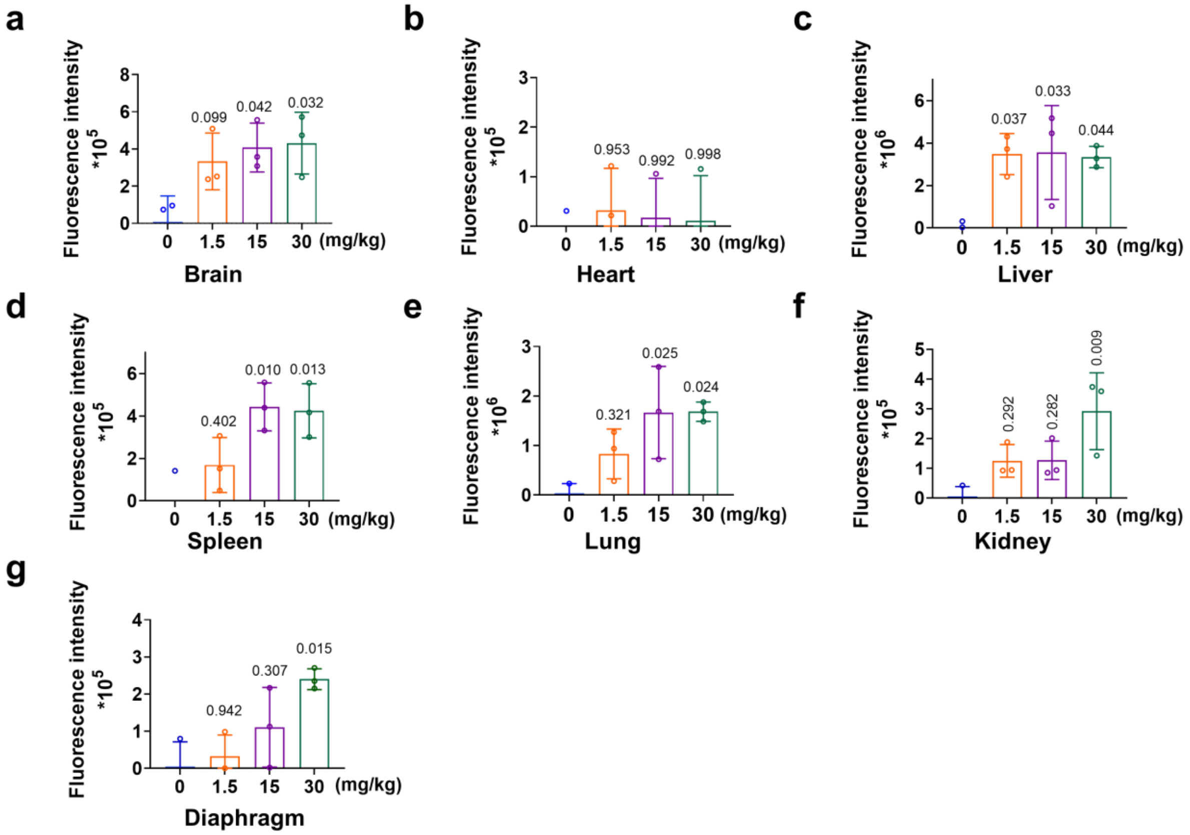

Supplementary Figure 19 Fluorescence intensity analysis of leaked EBD in mouse tissues. For evaluation of the fluorescence intensity of leaked EBD, EvolutionCapt software was used. All data are represented as the mean ± SD (n = 3 biologically independent animals). *P* values comparing with control were derived from one-way ANOVA followed by Tukey's multiple comparison tests and inserted in the panels. Source data are provided as a Source Data file.

Supplementary Table 1. Size distribution and ζ-potential of nanoplasitics measured by DLS.

Samples	Size distribution (d.nm) per intensity (%)	PDI	ζ-potential (mV)
**PS (H_2O)**	62 ± 5.0	0.08 ± 0.05	-35.4 ± 1.9
**PS (ECM)**	72 ± 2.0	0.16 ± 0.05	-7.5 ± 0.5
**PMMA (H_2O)**	89 ± 1.3	0.14 ± 0.01	-24.8 ± 0.7
**PMMA (ECM)**	Peak one: 100 ± 1.9<br>Peak two: 4913 ± 260.9	0.20 ± 0.02	-19.2 ± 4.0
**NH_2-PS (H_2O)**	Peak one: 31 ± 0.6<br>Peak two: 3984 ± 253.4	0.29 ± 0.01	31.0 ± 1.3
**NH_2-PS (ECM)**	Peak one: 46 ± 14.2<br>Peak two: 4255 ± 805.1	0.28 ± 0.05	29.4 ± 1.4

Supplementary Table 2. Details of primary and secondary antibodies.

Antibody name	Sources	Catalogue numbers	Dilutions
Caspase-3 p17 monoclonal antibodies	Santa Cruz Biotechnology	sc-373730	1:1000
Bax monoclonal antibody	Santa Cruz Biotechnology	sc-20067	1:1000
Bcl-2 Polyclonal Antibody	Wanlei Biotechnology	WL01556	1:500
β-Actin polyclonal antibody	Sangon Biotechnology	D110001	1:2000
LC3I/II polyclone antibody	Sigma	L7543	1:3000
Caspase 9/p35/p10 polyclone antibodies	Proteintech	10380-1-AP	1:1000
PI3K polyclone antibodies	Wanlei Biotechnology	WL03380	1:1000
p-PI3K polyclone antibodies	Biosynthesis Biotechnology	bs-6417R	1:1000
AKT polyclone antibodies	Wanlei Biotechnology	WL0003b	1:1000
p-AKT polyclone antibodies	Wanlei Biotechnology	WLP001a	1:1000
Beclin-1 polyclone antibodies	Wanlei Biotechnology	WL02508	1:1000
Atg5 polyclone antibodies	Wanlei Biotechnology	WL02411	1:1000
p62 polyclone antibodies	Wanlei Biotechnology	WL02385	1:1000
VE-Cadherin polyclone antibodies	Wanlei Biotechnology	WL02033	1:1000
Phospho-VE-Cadherin (Tyr658) polyclone antibodies	Affinity	AF8206	1:1000
Phospho-VE-Cadherin (Y731) polyclone antibodies	ImmunoWay	YP0808	1:1000
VE-Cadherin polyclone antibodies	Abcam	ab33168	1:400
Donkey anti-rabbi Alex 594 secondary antibody	Abcam	ab150076	1:500
α-Tubulin polyclone antibodies	Proteintech	11224-1-AP	1:2000
Biotinylated goat anti-rabbit IgG (H+L)	Beyotime Biotechnology	A0277	1:2000
Biotinylated goat anti-mouse IgG (H+L)	Beyotime Biotechnology	A0286	1:2000
HRP-labeled streptavidin antibodies	Beyotime Biotechnology	A0308	1:5000

Supplementary Table 3. Sequence of all primers used in the RT-qPCR experiment.

Primers	Forward	Reverse
Atg5	5′-AAAGATGTGCTTCGAGATGTGT-3′	5′-CACTTTGTCAGTTACCAACGTCA-3′
Beclin-1	5′-GGTGTCTCTCGCAGATTCATC-3′	5′-TCAGTCTTCGGCTGAGGTTCT-3′
p62	5′-AAGCCGGGTGGGAATGTTG-3′	5′-CCTGAACAGTTATCCGACTCCAT-3′
Bax	5′-CCCGAGAGGTCTTTTTCCGAG-3′	5′-CCAGCCCATGATGGTTCTGAT-3′
Bcl-2	5′-CCCAGAGTTTGAGCCGAGTG-3′	5′-CCCATCCCTTCGTCGTCCT-3′
GRP78	5′-CATCACGCCGTCCTATGTCG-3′	5′-CGTCAAAGACCGTGTTCTCG-3′
CHOP	5′-GGAAACAGAGTGGTCATTCCC-3′	5′-CTGCTTGAGCCGTTCATTCTC-3′
β-actin	5′-CATGTACGTTGCTATCCAGGC-3′	5′-CTCCTTAATGTCACGCACGAT-3′
