# Supplementary material for: Anionic nanoplastic exposure induces endothelial leakiness
Source: Nat Commun. 2022 Aug 13;13:4757. doi: 10.1038/s41467-022-32532-5 (PMC9376074; doi:10.1038/s41467-022-32532-5)
Supplement: Supplementary file 3 — Reporting Summary [file 41467_2022_32532_MOESM3_ESM.pdf]

nature portfolio

Corresponding author(s): Pu Chun Ke

Last updated by author(s): Jul 30, 2022

Reporting Summary

Nature Portfolio wishes to improve the reproducibility of the work that we publish. This form provides structure for consistency and transparency in reporting. For further information on Nature Portfolio policies, see our Editorial Policies and the Editorial Policy Checklist.

Statistics

For all statistical analyses, confirm that the following items are present in the figure legend, table legend, main text, or Methods section.

n/a	Confirmed	
☐	☒	The exact sample size (*n*) for each experimental group/condition, given as a discrete number and unit of measurement
☐	☒	A statement on whether measurements were taken from distinct samples or whether the same sample was measured repeatedly
☐	☒	The statistical test(s) used AND whether they are one- or two-sided<br>*Only common tests should be described solely by name; describe more complex techniques in the Methods section.*
☒	☐	A description of all covariates tested
☒	☐	A description of any assumptions or corrections, such as tests of normality and adjustment for multiple comparisons
☐	☒	A full description of the statistical parameters including central tendency (e.g. means) or other basic estimates (e.g. regression coefficient) AND variation (e.g. standard deviation) or associated estimates of uncertainty (e.g. confidence intervals)
☐	☒	For null hypothesis testing, the test statistic (e.g. *F*, *t*, *r*) with confidence intervals, effect sizes, degrees of freedom and *P* value noted<br>*Give P values as exact values whenever suitable.*
☒	☐	For Bayesian analysis, information on the choice of priors and Markov chain Monte Carlo settings
☒	☐	For hierarchical and complex designs, identification of the appropriate level for tests and full reporting of outcomes
☒	☐	Estimates of effect sizes (e.g. Cohen's *d*, Pearson's *r*), indicating how they were calculated

Our web collection on statistics for biologists contains articles on many of the points above.

Software and code

Policy information about availability of computer code

Data collection	Mice tissue fluorescence data collected using EvolutionCapt software. Protein data bank (PDB) files were collected using the program of Molecules in action, LLC.
Data analysis	ImageJ 1.53c, Origin (v7.5), GraphPad Prism (v 9.3.1), Harmony High-Content Imaging and Analysis software from PerkinElmer, FlowJo_V10, Python, MATLAB_R2017a, pyMol, Visual Molecular Dynamics (VMD), and Grace were used to perform the data analysis. No custom code/ algorithm was used.

For manuscripts utilizing custom algorithms or software that are central to the research but not yet described in published literature, software must be made available to editors and reviewers. We strongly encourage code deposition in a community repository (e.g. GitHub). See the Nature Portfolio guidelines for submitting code & software for further information.

Data

Policy information about availability of data

All manuscripts must include a data availability statement. This statement should provide the following information, where applicable:

- Accession codes, unique identifiers, or web links for publicly available datasets
- A description of any restrictions on data availability
- For clinical datasets or third party data, please ensure that the statement adheres to our policy

The source data underlying the respective main text (Figs. 1-4, 6) and Supplementary Figures (Supplementary Figures 1, 2, 4, 5, 9-13, 18 and 19) are provided as Source Data File. Source data are provided with this paper. The source data for Fig. 5 and Supplementary Figures 14-17 were deposited in the webserver https://dlab.clemson.edu/research/NanoPlasticEL/.
The description data for s-dimer and x-dimer were deposited in Protein Data Bank (PDB) under the accession codes 3PPE and 4ZT1.

Field-specific reporting

Please select the one below that is the best fit for your research. If you are not sure, read the appropriate sections before making your selection.

☒ Life sciences ☐ Behavioural & social sciences ☐ Ecological, evolutionary & environmental sciences

For a reference copy of the document with all sections, see nature.com/documents/nr-reporting-summary-flat.pdf

Life sciences study design

All studies must disclose on these points even when the disclosure is negative.

Sample size	All our in vitro experiments described in this paper were performed with n = 3. A minimum of 3 independent repeats was required to evaluate statistical significance. The in vivo studies were performed with 3 mice per group in both nanoplastic groups and control groups while adhering to our approved Southwest University Animal Care and Use Committee standard protocols and minimizing the number of animals to still achieve statistical validity. Details regarding sample sizes and statistical tests of all experiments are provided in the Methods section and figure captions.
Data exclusions	We did not exclude any data.
Replication	The in vitro experiments were repeated for 3 times, and the results were consistent with each other. For the in vivo work, each group contained 3 mice.
Randomization	Randomization was relevant in our class in the mice experiments. But they were all aged and sex matched before randomization into the various treatment groups.
Blinding	The sub team analyzing the mouse data was independent from the sub team working on the mouse experiments and was blinded from the experimental conditions/groups. No data were excluded.

Reporting for specific materials, systems and methods

We require information from authors about some types of materials, experimental systems and methods used in many studies. Here, indicate whether each material, system or method listed is relevant to your study. If you are not sure if a list item applies to your research, read the appropriate section before selecting a response.

Materials & experimental systems

n/a	Involved in the study	
☐	☒	Antibodies
☐	☒	Eukaryotic cell lines
☒	☐	Palaeontology and archaeology
☐	☒	Animals and other organisms
☒	☐	Human research participants
☒	☐	Clinical data
☒	☐	Dual use research of concern

Methods

n/a	Involved in the study	
☒	☐	ChIP-seq
☐	☒	Flow cytometry
☒	☐	MRI-based neuroimaging

Antibodies

Antibodies used

Cleaved caspase 3 monoclonal antibody (Santa Cruz Biotechnology; Product number: sc-373730), 1:1000 dilution
Bax monoclonal antibody (Santa Cruz Biotechnology; Product number: sc-20067), 1:1000 dilution
Bcl-2 polyclonal antibody (Wanlei Biotechnology; Product number: WL01556), 1:500 dilution
β-Actin polyclonal antibody (Sangon Biotechnology; Product number: D110001), 1:2000 dilution
LC3I/II polyclone antibody (Sigma; Product number: L7543), 1:3000 dilution
Caspase 9/p35/p10 polyclone antibody (Proteintech; Product number: 10380-1-AP), 1:1000 dilution
PI3K polyclone antibody (Wanlei Biotechnology; Product number: WL03380), 1:1000 dilution
p-PI3K polyclone antibody (Biosynthesis Biotechnology; Product number: bs-6417R), 1:1000 dilution
AKT polyclone antibody (Wanlei Biotechnology; Product number: WL0003b), 1:1000 dilution
p-AKT polyclone antibody (Wanlei Biotechnology; Product number: WLP001a), 1:1000 dilution
Beclin-1 polyclone antibody (Wanlei Biotechnology; Product number: WL02508), 1:1000 dilution
Atg5 polyclone antibody (Wanlei Biotechnology; Product number: WL02411), 1:1000 dilution
p62 polyclone antibody (Wanlei Biotechnology; Product number: WL02385), 1:1000 dilution
VE-Cadherin polyclone antibody (Wanlei Biotechnology; Product number: WL02033), 1:1000 dilution
Phospho-VE-Cadherin (Tyr658) polyclone antibody (Affinity; Product number: AF8206), 1:1000 dilution
Phospho-VE-Cadherin (Y731) polyclone antibody (ImmunoWay; Product number: YP0808), 1:1000 dilution

VE-Cadherin polyclone antibody (Abcam; Product number: ab33168), 1:400 dilution
Donkey anti-rabbi Alex 594 secondary antibody (Abcam; Product number: ab150076), 1:500 dilution
α-Tubulin polyclone antibody (Proteintech; Product number: 11224-1-AP), 1:2000 dilution
Biotinylated goat anti-rabbit IgG (H+L) (Beyotime Biotechnology; Product number: A0277), 1:2000 dilution
Biotinylated goat anti-mouse IgG (H+L) (Beyotime Biotechnology; Product number: A0286), 1:2000 dilution
HRP-labeled streptavidin antibody (Beyotime Biotechnology; Product number: A0308), 1:5000 dilution

Validation	Validation of each antibody was performed under information offered by each supplier.

Eukaryotic cell lines

Policy information about cell lines

Cell line source(s)	Human umbilical vein endothelial cells (HUVEC, ATCC, USA, Cat# CRL-1730) was used in this study.
Authentication	We did not authenticate the cell lines because they were all from commercial sources and they had their own individual companies' quality control policies.
Mycoplasma contamination	We did not test for mycoplasma contamination because the source company had already tested for mycoplasma.
Commonly misidentified lines (See ICLAC register)	None

Animals and other organisms

Policy information about studies involving animals; ARRIVE guidelines recommended for reporting animal research

Laboratory animals	Eighteen 10-week-old male Swiss mice, three 8-month-old male Large White Swines and three 4-month-old male New Zealand Rabbits were used.
Wild animals	No wild animals were used in the study.
Field-collected samples	No field collected samples were used in the study.
Ethics oversight	Southwest University Animal Care and Use Committee.

Note that full information on the approval of the study protocol must also be provided in the manuscript.

Flow Cytometry

Plots

Confirm that:

- [x] The axis labels state the marker and fluorochrome used (e.g. CD4-FITC).
- [x] The axis scales are clearly visible. Include numbers along axes only for bottom left plot of group (a 'group' is an analysis of identical markers).
- [ ] All plots are contour plots with outliers or pseudocolor plots.
- [ ] A numerical value for number of cells or percentage (with statistics) is provided.

Methodology

Sample preparation	Approximately 1 × 106 cells/well were grown on 6-well plates and exposed to the PS nanoplastic (0.05, 0.1, 0.25 and 0.5 mg/mL) for 1, 3 or 6 h. Cells in the control group received serum-free medium treatment for 1, 3 or 6 h and were then incubated with DCFH-DA (Sigma-Aldrich, USA) indicator in the dark at 37 °C for 30 min. Cells were washed twice with phosphate buffered saline (PBS).<br>Approximately 1 × 106 cells/well were seeded in 6-well plates overnight followed by the treatment of PS nanoplastic or NH2-PS nanoplastic at 0.05 or 0.5 mg/mL for 1, 3 or 6 h. Then, cells were washed with PBS and analyzed using a BD FACS MelodyTM flow cytometry.<br>A total of 1 × 104 events was acquired for each sample from three independent experiments.
Instrument	BD FACS MelodyTM flow cytometer
Software	FlowJo_V10
Cell population abundance	>10000 cells were recorded for the desired population

Gating strategy	FACS gating strategy for measuring the ROS value of HUVECs via the FITC channel. Gating for cells incubated with DCFH-DA indicator. FACS gating strategy for measuring the fluorescence intensity of PS nanoplastic or NH2-PS nanoplastic in HUVECs via the FITC channel.

☒ Tick this box to confirm that a figure exemplifying the gating strategy is provided in the Supplementary Information.
